# Retraction: Role of Activated Rac1/Cdc42 in Mediating Endothelial Cell Proliferation and Tumor Angiogenesis in Breast Cancer

**DOI:** 10.1371/journal.pone.0240575

**Published:** 2020-10-08

**Authors:** The PLOS ONE Editors

After this article [1] and its subsequent correction [2] were published, concerns were raised about results reported in Figs 1 and 2.

Specifically:

- In Fig 1B, the pCEFL-GST and pCEFL-GST+MG132 panels appear to report the same image.
- Similarities were noted between the following western blot results reported in Fig 2 of [1] and Fig 1 of [3]. In each case, the similar data represent results for different experiments and/or samples in the two articles.
  ○ Fig 2D VEGF in [1] appears similar to Fig 1A Ndrg2 blot lanes 1–2 in [3], flipped horizontally.
  ○ Fig 2E VEGF in [1] appears similar to Fig 1A Ngrd2 blot lanes 3–5 in [3], flipped horizontally.
  ○ Fig 2E β-actin in [1] appears similar to Fig 1A β-actin blot lanes 3–5 in [3], flipped horizontally.

In response to the concerns about similarities between results reported in [1] and [3], the corresponding author noted that in looking into this issue they found errors in data collection and sorting. The original data underlying the western blot results are no longer available, and as such the above issues cannot be clarified. The corresponding author requested retraction of the article.

In light of the unresolved concerns and in line with the corresponding author’s request, the *PLOS ONE* Editors retract this article.

YX agreed with retraction. The other authors either could not be reached or did not respond directly.
